# Liver Cancer Etiology in Asian Subgroups and American Indian, Black, Latino, and White Populations

**DOI:** 10.1001/jamanetworkopen.2025.2208

**Published:** 2025-03-27

**Authors:** Paulo S. Pinheiro, Juanjuan Zhang, Veronica Wendy Setiawan, Hannah M. Cranford, Robert J. Wong, Lihua Liu

**Affiliations:** 1 Division of Epidemiology & Population Health Sciences, Department of Public Health Sciences, University of Miami School of Medicine, Miami, Florida; 2 Sylvester Comprehensive Cancer Center, University of Miami Health System, Miami, Florida; 3 Los Angeles Cancer Surveillance Program, Department of Population and Public Health Sciences, Keck School of Medicine, University of Southern California, Los Angeles; 4 Department of Population and Public Health Sciences, Keck School of Medicine, University of Southern California, Los Angeles; 5 Division of Gastroenterology and Hepatology, Stanford University School of Medicine, Palo Alto, California; 6 Gastroenterology Section, Veterans Affairs Palo Alto Health Care System, Palo Alto, California

## Abstract

**Importance:**

Hepatocellular carcinoma (HCC) is a major public health issue in the US, linked to hepatitis C virus (HCV), hepatitis B virus (HBV), alcohol-related liver disease (ALD), and metabolic dysfunction–associated steatotic liver disease (MASLD). Monitoring HCC high-risk populations and trends across increasingly diverse groups is crucial for targeted prevention and intervention strategies.

**Objective:**

To evaluate incidence patterns and temporal trends in HCC incidence by etiology across sex, race and ethnicity, and detailed Asian subgroups in California, highlighting emerging metabolic risks and declining viral influences, to guide targeted prevention strategies.

**Design, Setting, and Participants:**

This population-based, retrospective cohort study used data from the California Cancer Registry linked to statewide hospital diagnostic records for etiology classification. All HCC cases diagnosed between 2010 and 2018 in California were included. Data were analyzed from March 28 to November 3, 2024.

**Exposure:**

Etiology of HCC, categorized as HCV, HBV, ALD, MASLD, or other causes.

**Main Outcomes and Measures:**

Age-adjusted incidence rates (AAIRs) of HCC, stratified by sex, race and ethnicity, and etiology. Trends were analyzed using Joinpoint regression.

**Results:**

From 2010 to 2018, 31 671 patients (23 558 [74.4%] male; median [IQR] age, 64 [15] years) were newly diagnosed with HCC in California, with 14 664 (46.3%) due to HCV, 7457 (23.5%) due to MASLD, 3941 (12.4%) due to ALD, and 3271 (10.3%) due to HBV. By 2017 to 2018, MASLD accounted for 27.4% of HCCs, surpassing HCV among women. HCV-related HCC rates per 100 000 population were highest among American Indian (men: 12.8; women: 3.6), Black (men: 10.8; women: 3.0), and US-born Latino (men: 15.7; women: 3.5) populations and specific Asian groups (particularly Cambodian [men: 15.5; women: 6.3] and Vietnamese [men: 13.7; women: 4.8]). Rates of MASLD-related HCC per 100 000 population were highest among American Indian (men: 4.2; women: 2.7), Asian and Pacific Islander (men: 3.9; women: 1.8), and Latino (men: 4.4; women: 2.9) populations. Rates of HBV-related HCC per 100 000 population remained high throughout the study period in some Asian and Pacific Islander subgroups (Cambodian [men: 18.3; women: 3.4], Chinese [men: 10.1; women: 2.6], Korean [men: 11.5; women: 2.8], Laotian [men: 25.0; women: 5.4], and Vietnamese [men: 16.6; women: 3.5]), but not all subgroups, and HBV-related HCC declined overall. During 2014 to 2018, the overall HCC incidence changed annually by −3.1% (95% CI −4.8% to −1.4%) in men and −3.2% (95% CI, −10.9% to −0.9%) in women, largely due to declines in HCV-related HCC. However, MASLD- and ALD-related HCC rates increased throughout 2010 to 2018 (MASLD: 1.9% [95% CI, 0.8% to 3.0%]; ALD: 1.9% [95% CI, 0.6% to 3.1%]).

**Conclusions and Relevance:**

This retrospective cohort study found that etiology of HCC was associated with detailed racial and ethnic groups, with notable variation across disaggregated Asian groups. Rates of MASLD-related and ALD-related HCC were increasing, posing new challenges for prevention. American Indian, Asian and Pacific Islander, and Latino populations were disproportionately affected by HCC, beyond viral causes. Targeted public health interventions addressing alcohol, viral, and metabolic risk factors are needed.

## Key Points

**Question:**

What are etiologies of hepatocellular carcinoma (HCC) among detailed racial and ethnic populations in the US, and how heterogeneous are Asian groups regarding HCC risk?

**Findings:**

This cohort study including 31 671 patients with HCC in California found that rates of HCC were highest among American Indian, Latino, and Southeast Asian (especially Cambodian and Vietnamese) populations. Etiologies differed by group and time, with viral-related HCC declining and HCC linked to metabolic dysfunction–associated steatotic liver disease and alcohol increasing.

**Meaning:**

These findings highlight the need for continued surveillance and targeted public health strategies addressing viral and increasing metabolic risk factors in diverse populations.

## Teaser

This cohort study examines incidence patterns and temporal trends in hepatocellular carcinoma incidence by etiology across sex, race and ethnicity, and detailed Asian subgroups in California.

## Introduction

Liver cancer is projected to cause nearly 30 000 deaths in the US in 2024,^1^ with hepatocellular carcinoma (HCC) as the most common and highly fatal form, carrying an 18% 5-year survival rate.^2^ The primary drivers of HCC—chronic infections with hepatitis C virus (HCV) and hepatitis B virus (HBV), alcohol-related liver disease (ALD), and metabolic dysfunction–associated liver disease (MASLD)—account for approximately 90% of HCCs. Since these risk factors are largely modifiable, HCC incidence and mortality are potentially preventable.^3,4,5^

HCC incidence rates in the US are highest among American Indian people, followed by Asian and Hispanic and Latino populations.^6,7^ A recent study in Florida found HCC incidence was associated with specific etiologies in detailed Latino and non-Latino Black populations.^8^ However, in California—a state with even greater racial and ethnic diversity—population-based analyses of HCC etiology remain underexplored. California’s population includes Latino individuals (primarily of Mexican descent [US-born and foreign-born]), non-Latino Black and White individuals, and a uniquely diverse array of Asian and Pacific Islander communities, such as Chinese, Filipino, Hmong, Japanese, Korean, Laotian, and South Asian. Each group possesses distinct cultural, genetic, and socioeconomic factors influencing health outcomes, including HCC risk.^9,10^

Both nationally and in California, Asian populations exhibit high rates of liver cancer; yet notable differences exist among detailed groups.^10,11,12^ For instance, in the US, Korean and Vietnamese populations have a higher prevalence of chronic HBV infection compared with other groups.^13^ Patterns of alcohol use also vary significantly, being lower among Chinese individuals but relatively higher among Japanese and Korean individuals.^14^ Additionally, while Asian individuals in the US tend to experience a high prevalence of MASLD, this often occurs despite lower rates of obesity and comorbidities.^15^

Despite the heterogeneity of risk factors, certain known patterns are consistent across populations. HCV-related HCC disproportionately affects individuals born between 1945 and 1965, regardless of race or ethnicity.^16,17,18,19^ Elevated HCC rates among Asian populations are predominantly tied to chronic HBV infection.^20^ Yet, population-based data exploring these diverse groups remain scarce. In California alone, approximately 2% of the population lives with HBV or HCV,^21^ and 7.1% report heavy alcohol consumption.^22^

Recent advances in therapeutics and screening guidelines have the potential to influence HCC rates. Sustained virologic response to direct-acting antivirals (DAAs), introduced in 2013, has been shown to substantially reduce HCC risk.^23,24,25^ Similarly, effective management of chronic HBV infection has demonstrated protective effects.^26,27,28^ However, despite recommendations from the Centers for Disease Control and Prevention for at least 1 lifetime screening for HCV and HBV in adults,^26,29^ screening remains underused,^27,28,30^ and many individuals with viral hepatitis go untreated.^31^

Variations across detailed racial and ethnic groups underscore the need for tailored prevention strategies that address the unique risk factors shaping health outcomes in these populations. To address this knowledge gap, this study leverages newly available data from the California Cancer Registry (CCR), linked with hospital diagnostic records. Recognized for its quality and completeness by the North American Association of Central Cancer Registries,^32^ the CCR provides a robust foundation for analyzing HCC patterns and trends from 2010 to 2018. This analysis seeks to uncover incidence rates by HCC etiology across racial and ethnic groups and among Asian and Pacific Islander subgroups, offering a nuanced understanding of evolving patterns to inform prevention efforts.

## Methods

This cohort study was approved by the California Cancer Registry and the California Health and Human Services Agency Committee for the Protection of Human Subjects. Informed consent was not required, as the study used deidentified data. This study was conducted and reported in accordance with the Strengthening the Reporting of Observational Studies in Epidemiology (STROBE) reporting guideline.

### Study Population

All incidents of invasive liver cancer reported to the CCR diagnosed between 2010 and 2018 were included in this study. Eligible HCCs comprised all *International Classification of Disease for Oncology, Third Edition* (*ICD-O-3*), HCC morphologies (8170-8180) (29 560 patients [93.3%]), and cancers coded as C22.0 (liver) with morphologies 8000 to 8010 (2111 patients [6.7%]) that did not have a primary cancer (eg, breast, colon) preceding the HCC diagnosis to exclude potentially metastatic cancers originating from other primary sites.

### Data Linkage and Etiology Categories

The CCR routinely links (on an annual basis) all cancer cases with data from the California Department of Health Care Access and Information (HCAI), mainly for follow-up purposes. However, the CCR-HCAI linked data can also be used to obtain diagnosis codes of precancer medical conditions for every medical episode (hospital inpatient diagnostic, emergency department encounters, and ambulatory surgery center use). The linkage is conducted by CCR and is primarily deterministic but also incorporates probabilistic methods based on similar Social Security numbers and year, month, or day of birth; as well as similarity in zip code, sex, and a Levenstein distance of 2 or less for Social Security numbers together with similarity in date of birth, sex, and zip code. For this study, diagnoses of HCC-related conditions from HCAI within 5 years prior to HCC diagnosis were classified based on previous studies^33,34,35,36^ (eTable 1 in Supplement 1) and used to identify 7 categories of conditions associated with HCC, including HCV, HBV, HCV and HBV, ALD, MASLD, and rarer causes: autoimmune diseases and genetic causes (eg, hemochromatosis). An eighth category, cryptogenic, was defined as HCCs that matched with diagnostic data but lacked any code for the other 7 categories. In cases of overlap, a predominant-cause category was selected using the hierarchical approach by Beste et al,^37^ as previously described,^36^ based on the decreasing strength of association for each HCC etiology.

### Statistical Analyses

#### Additional Covariates and Imputation Methods

We computed proportions of individuals with HCC by etiology for each racial and ethnic group, sex, age group, stage at diagnosis, socioeconomic status (SES), nativity (US-born vs born outside the US), region of residence, insurance type, and presence of cirrhosis. The SES measure for each patient was derived from the CCR SES index, which is based on the census block group of residence.^38^ This index is categorized into quintiles and incorporates detailed demographic, social, economic, and housing data from the American Community Survey.^39^ We compared distributions for each characteristic using χ^2^ tests. Level I race and ethnicity includes broad racial and ethnic groups (American Indian, Asian or Pacific Islander, Latino/a, non-Latino Black, and non-Latino White). For simplicity, we use the terms *White population* and *Black population* only, with the understanding that these refer specifically to non-Latino populations. Level II race and ethnicity includes detailed groups: Latino individuals born in the US and born elsewhere and, within the Asian and Pacific Islander population, Chinese, Filipino, Japanese, Korean, Pacific Islander (including Hawaiian), South Asian (Bangladeshi, Indian, Nepalese, Pakistani, and Sri Lankan), Vietnamese, and other Southeast Asian (Cambodian, Hmong, Laotian, and Thai), defined according to the NHAPIIA algorithm.^40^ A category for Asian people not otherwise specified includes other specific Asian groups (eg, Burmese, Indonesian, Mongolian) and Asian people with unknown specific race. The final percentage of individuals in this group was 3.1% of 6561 Asian and Pacific Islander patients, likely representing a very small part of the specific Asian groups studied.

To avoid underestimating etiology-specific counts, which could distort rates and trends, we performed multiple imputations with 20 iterations based on sex, racial and ethnic group, age group, region of residence (defined by CCR as northern, southern, and inland) (eTable 2 in Supplement 1), and year of diagnosis for patients who did not initially match with any data sources for etiology (4858 patients [15.3%]). These individuals were imputed into 8 mutually exclusive cause groups: HCV, HBV, HCV and HBV, ALD, MASLD, cryptogenic, genetic, and autoimmune. For nativity (birth within the US or its territories vs born elsewhere), we imputed missing information for 5898 individuals (18.6% total; 18.2% among Latino populations), to enable reporting on the important differences in HCC risk between Latinos born in the US vs elsewhere, documented in prior studies.^8,41^ Lastly, to assess the robustness of our findings, we conducted sensitivity analyses by simulating extreme but plausible scenarios. For etiology, we examined the impact of varying the proportion of HCV-related HCCs (the most common etiology) among those with missing etiology by ±10 percentage points while maintaining proportional allocations for other causes. For nativity among Latino patients, we explored 2 opposing scenarios: all patients with missing nativity assigned as US-born and all patients assigned as born elsewhere.

#### Incidence Rates and Trends in HCC by Etiology

To demonstrate population-level differences, we calculated annualized, sex-stratified, etiology-specific age-adjusted incidence rates (AAIRs) for HCC for each level I and II race and ethnicity group using the 2000 US standard population for the entire 2010 to 2018 period. Joint viral etiologies (HBV and HCV) were divided proportionately based on the distribution of HBV and HCV HCCs for each level I and II racial group, by sex and age group. Population denominators corresponding to each age group, race and ethnicity, and sex came from the American Community Survey for 2010 to 2018.^42^ Finally, we used Joinpoint regression^43^ to assess AAIRs of the specific HCC cause by age group (all races and sexes combined) in young adults (age ≤49 years), middle-aged adults (age 50-69 years) and older adults (age ≥70 years); by sex; and by race and ethnicity (level I) with AAIRs listed for 2010 to 2011 and the most recent period available (2017-2018). For the study of trends, the partition of combined HBV and HCV HCC respected sex, age group, and year of diagnosis. We also analyzed the effect of the 1945 to 1965 birth cohort on HCV-related HCC rates by race and ethnicity (level I) and by US-born vs born elsewhere for Latino people. All numbers presented in tables are postimputation unless otherwise described.

The threshold for statistical significance for all analyses was set at *P* ≤ .05. Data were analyzed using SAS statistical software version 9.4 (SAS Institute) and SPSS statistical software version 22.0 (IBM). Data were analyzed from March 28 to November 3, 2024. 

## Results

### Distribution of HCC Etiologies

In this retrospective cohort study, we analyzed all 31 671 patients (23 558 [74.4%] male; median [IQR] age, 64 [15] years) with HCC diagnosed in California from 2010 to 2018. Table 1 summarizes the distribution of HCC etiologies by demographics. Over the study period, the most common related condition was HCV (14 664 patients [46.3%]), followed by MASLD (7457 patients [23.5%]), ALD (3941 patients [12.4%]), and HBV (3271 patients [10.3%]). Notably, by 2017 to 2018, the proportion of HCC related to HCV decreased to 42.5%, while the proportion for MASLD increased to 27.4%.

**Table 1.** Sociodemographic and Clinical Characteristics by Predominant Cause of HCC

Characteristic	Total, No. (column %)^a^	No. (row %)				*P* value^b^
		HCV	MASLD	ALD	HBV	
Total patients	31 671 (100)	14 664 (46.3)	7457 (23.5)	3941 (12.4)	3271 (10.3)	NA
Age, y						
Median (IQR)	64 (15)	61	73	65	62	NA
<49	1649 (5.2)	629 (38.1)	174 (10.5)	171 (10.3)	412 (25.0)	<.001
50-69	19 705 (62.2)	11 464 (58.2)	2586 (13.1)	2422 (12.3)	1989 (10.1)	
≥70	10 317 (32.6)	2572 (24.9)	4697 (45.5)	1348 (13.1)	870 (8.4)	
Stage at diagnosis						
Localized	15 583 (49.2)	7615 (48.9)	3344 (21.5)	1827 (11.7)	1735 (11.1)	<.001
Regional	7396 (23.4)	3534 (47.8)	1635 (22.1)	948 (12.8)	769 (10.4)	
Distant	4124 (13.0)	1751 (42.5)	1075 (26.1)	536 (13.0)	424 (10.3)	
Unknown	4568 (14.4)	1765 (38.6)	1402 (30.7)	631 (13.8)	342 (7.5)	
Socioeconomic status						
Lowest	7328 (23.1)	3689 (50.3)	1610 (22.0)	1056 (14.4)	511 (7.0)	<.001
Lower-middle	7398 (23.4)	3614 (48.9)	1643 (22.2)	923 (12.5)	704 (9.5)	
Middle	6762 (21.4)	3176 (47.0)	1582 (23.4)	821 (12.1)	675 (10.0)	
Upper-middle	5808 (18.3)	2516 (43.3)	1476 (25.4)	646 (11.1)	707 (12.2)	
Highest	4375 (13.8)	1669 (38.2)	1146 (26.2)	496 (11.3)	675 (15.4)	
Nativity status						
US birth	19 292 (60.9)	10 765 (55.8)	4227 (21.9)	2354 (12.2)	553 (2.9)	<.001
Born elsewhere	12 379 (39.1)	3899 (31.5)	3230 (26.1)	1587 (12.8)	2718 (22.0)	
Presence of cirrhosis as per diagnostic code data^c^						
Code present	18 603 (69.4)	10 669 (57.4)	2827 (15.2)	2838 (15.3)	1582 (8.5)	<.001
Code not present	8210 (30.6)	1989 (24.2)	3652 (44.5)	486 (5.9)	820 (10.0)	
Sex						
Male	23558 (74.4)	11 336 (48.1)	4611 (19.6)	3442 (14.6)	2583 (11.0)	<.001
Female	8113 (25.6)	3328 (41.0)	2846 (35.1)	499 (6.1)	688 (8.5)	
Race and ethnicity						
American Indian	413 (1.3)	240 (58.0)	75 (18.2)	67 (16.2)	NA	<.001
Asian and Pacific Islander	6561 (20.7)	1719 (26.2)	1541 (23.5)	252 (3.8)	2551 (38.9)	
Latino	9992 (31.5)	4724 (47.3)	2500 (25.0)	1884 (18.9)	224 (2.2)	
Non-Latino Black	2419 (7.6)	1639 (67.7)	359 (14.8)	171 (7.1)	124 (5.1)	
Non-Latino White	12 190 (38.5)	6314 (51.8)	2969 (24.4)	1561 (12.8)	356 (2.9)	
Other^d^	96 (0.3)	29 (29.9)	13 (13.5)	NA	NA	
Detailed Asian and Pacific Islander subgroup						
Chinese	1713 (24.4)	249 (14.5)	320 (18.7)	27 (1.6)	970 (56.6)	<.001
Filipino	1167 (16.6)	188 (16.1)	527 (45.2)	77 (6.6)	286 (24.5)	
Japanese	305 (4.3)	116 (37.9)	127 (41.6)	25 (8.2)	20 (6.6)	
Korean	628 (9.0)	151 (24.0)	103 (16.4)	22 (3.5)	311 (49.6)	
Pacific Islander	201 (2.9)	73 (36.2)	56 (27.9)	17 (8.5)	41 (20.3)	
South Asian^e^	257 (3.7)	94 (36.7)	92 (35.8)	26 (10.1)	29 (11.4)	
Vietnamese	1634 (23.3)	511 (31.3)	220 (13.5)	29 (1.8)	604 (37.0)	
Other Southeastern Asian^f^						
Overall	455 (6.5)	125 (27.5)	69 (15.1)	24 (5.3)	200 (44.0)	
Cambodian	193 (2.8)	72 (37.1)	31 (16.1)	12 (6.3)	31 (16.1)	
Hmong	47 (0.7)	NA	NA	NA	NA	
Laotian	128 (1.8)	29 (22.9)	16 (12.9)	NA	16 (12.9)	
Thai	87 (1.2)	19 (22.3)	15 (17.3)	NA	15 (17.3)	
Other	201 (2.9)	213 (105.7)	27 (13.4)	NA	89 (44.4)	
Detailed Latino group						
US-born	5097 (51.0)	2963 (58.1)	1069 (21.0)	692 (13.6)	82 (1.6)	<.001
Born elsewhere	4895 (49.0)	1761 (36.0)	1431 (29.2)	1193 (24.4)	142 (2.9)	
Region						
Northern California	11711 (37.0)	5560 (47.5)	2471 (21.1)	1275 (10.9)	1529 (13.1)	<.001
Southern California	13756 (43.4)	6079 (44.2)	3394 (24.7)	1818 (13.2)	1452 (10.6)	
Inland California	6204 (19.6)	3025 (48.8)	1592 (25.7)	848 (13.7)	290 (4.7)	

Abbreviations: ALD, alcoholic liver disease; HBV, hepatitis B virus; HCC, hepatocellular carcinoma; HCV, hepatitis C virus; MASLD, metabolic dysfunction–associated steatotic liver disease; NA, not available (small numbers of observations are not shown due to confidentiality protection).

^a^ Includes all listed as well as cryptogenic and others (eg, genetic, autoimmune).

^b^ *P* value from χ^2^ test of independence, computed on 4 categories of cause-specific HCC.

^c^ Cirrhosis has multiple causes and was not considered an etiology. These data were based on nonimputed cases only (N = 26 813).

^d^ Includes all other patients without a listed group or those with an unknown classification.

^e^ Includes all patients of races related to Bangladesh, India, Nepal, Pakistan, and Sri Lanka.

^f^ Includes all patients of races related to Cambodia, Laos, and Thailand, plus Hmong people.

By race and ethnicity, there were 413 American Indian patients (1.3%), 6561 Asian and Pacific Islander patients (20.7%), 2419 Black patients (7.6%), 9992 Latino patients (31.5%), and 12 190 White patients (38.5%). Among 6561 Asian and Pacific Islander patients, there were 193 Cambodian patients (2.9%), 1713 Chinese patients (26.1%), 1167 Filipino patients (17.8%), 47 Hmong patients (0.7%), 305 Japanese patients (4.6%), 628 Korean patients (9.6%), 128 Laotian patients (2.0%), 201 Pacific Islander patients (3.1%), 257 South Asian patients (3.9%), 87 Thai patients (1.3%), 1634 Vietnamese patients (24.9%), and 201 patients with other Asian race and ethnicity (3.1%). HCV-related HCC rates were higher among men, US-born people, and those from lower SES groups, whereas HBV-related HCC was more common among higher SES groups. The stage at diagnosis was slightly more favorable for HCV-related HCC and HBV-related HCC compared with ALD-related HCC and MASLD-related HCC. Additionally, Cambodian and Hmong groups had the highest proportions of Medicaid coverage (eTable 3 and eTable 4 in Supplement 1). Cirrhosis, often underreported in population-based HCC studies,^8^ was most prevalent among patients with HCV-related HCC and ALD-related HCC.

### Incidence Rates by Race and Ethnicity, Sex, and Etiology

Table 2 presents AAIRs by sex, etiology, and racial and ethnic groups. HCC rates were 12.9 (95% CI, 12.8-13.1) events per 100 000 population for males and 4.0 (95% CI, 3.9-4.1) events per 100 000 population for females, with considerable variation across populations. The most extreme HCC rates, encompassing both the highest and lowest values, were observed in specific Asian and Pacific Islander subpopulations, while Black, Latino, and White populations exhibited intermediate rates across the spectrum. For instance, in males, rates ranged from 7.3 (95% CI, 6.2-8.5) events per 100 000 population among Japanese men to 48.6 (95% CI, 39.4-59.0) events per 100 000 population among Cambodian men, and for females, rates from 2.4 (95% CI, 1.8-3.2) events per 100 000 population in South Asian women to 14.0 (95% CI, 10.6-18.2) events per 100 000 population in Cambodian women.

**Table 2.** AAIRs by Etiology by Detailed Race and Ethnicity and Sex

Race and ethnicity	No.	Foreign-born, %	HCC AAIR (95% CI), per 100 000 population				
			Total^a^	HCV	MASLD	ALD	HBV
**Males**							
Overall^b^	23 558	8670 (36.8)	12.9 (12.8-13.1)	5.8 (5.6-5.9)	2.9 (2.8-3.0)	1.9 (1.9-2.0)	1.5 (1.4-1.5)
American Indian	303	13 (4.0)	23.5 (20.7-26.5)	12.8 (10.9-14.8)	4.2 (2.9-5.8)	4.8 (3.6-6.3)	0.3 (0.1-0.9)
Asian and Pacific Islander							
Overall^c^	4716	4335 (91.9)	17.1 (16.6-17.6)	3.6 (3.3-3.8)	3.9 (3.7-4.2)	0.8 (0.7-1.0)	7.5 (7.2-7.8)
Chinese	1246	1181 (94.8)	16.0 (15.1-16.9)	1.5 (1.2-1.8)	2.8 (2.4-3.2)	0.3 (0.2-0.5)	10.1 (9.4-10.8)
Filipino	792	731 (92.3)	13.7 (12.8-14.8)	1.7 (1.4-2.1)	6.2 (5.5-6.9)	1.2 (0.9-1.5)	3.6 (3.2-4.1)
Japanese	159	64 (40.3)	7.3 (6.2-8.5)	2.4 (1.8-3.2)	2.8 (2.1-3.6)	1.1 (0.7-1.6)	0.6 (0.3-1.1)
Korean	432	419 (97.0)	19.8 (17.9-21.8)	3.1 (2.3-3.9)	2.9 (2.1-3.7)	1.0 (0.6-1.5)	11.5 (10.1-13.0)
Pacific Islander	142	51 (35.9)	19.0 (15.8-22.7)	5.9 (4.3-8.0)	6.0 (4.1-8.5)	2.1 (1.1-3.6)	4.0 (2.7-5.7)
South Asian^d^	199	196 (98.5)	8.4 (7.2-9.8)	2.5 (1.9-3.2)	3.5 (2.6-4.5)	1.0 (0.6-1.4)	1.1 (0.7-1.6)
Vietnamese	1280	1269 (99.1)	40.1 (37.9-42.5)	13.7 (12.4-15.1)	5.8 (4.9-6.8)	0.9 (0.6-1.3)	17.4 (16.0-19.0)
Other Southeastern Asian							
Overall	332	325 (97.9)	36.1 (32.0-40.6)	7.8 (5.9-10.1)	6.2 (4.3-8.5)	2.5 (1.5-3.8)	16.6 (14.1-19.5)
Cambodian	133	132 (99.3)	48.6 (39.4-59.0)	15.5 (10.1-22.4)	NA	NA	18.3 (13.6-24.0)
Hmong	34	33 (97.1)	20.8 (13.8-29.7)	NA	NA	NA	10.0 (5.7-16.1)
Laotian	104	100 (96.2)	46.1 (37.3-56.3)	8.6 (5.0-13.6)	NA	NA	25.0 (18.9-32.5)
Thai	61	60 (98.4)	33.2 (22.0-47.7)	NA	NA	NA	14.8 (8.7-23.8)
Latino							
Overall^c^	7268	3303 (45.4)	18.2 (17.7-18.6)	7.9 (7.7-8.2)	4.4 (4.2-4.7)	4.4 (4.2-4.6)	0.3 (0.3-0.4)
US-born	3965	0	27.9 (27.0-28.9)	15.7 (15.0-16.3)	5.8 (5.3-6.3)	4.8 (4.4-5.2)	0.2 (0.2-0.3)
Born elsewhere	3303	3303 (100)	13.3 (12.8-13.8)	4.1 (3.9-4.4)	3.6 (3.4-3.9)	4.2 (3.9-4.5)	0.4 (0.3-0.5)
Non-Latino Black	1816	103 (5.7)	16.4 (15.6-17.2)	10.8 (10.2-11.4)	2.5 (2.2-2.9)	1.3 (1.1-1.6)	0.9 (0.7-1.1)
Non-Latino White	9381	907 (9.7)	9.2 (9.1-9.4)	4.8 (4.7-4.9)	2.1 (2.1-2.2)	1.4 (1.3-1.4)	0.3 (0.2-0.3)
**Females**							
Overall^b^	8113	3709 (45.7)	4.0 (3.9-4.1)	1.6 (1.5-1.6)	1.4 (1.4-1.5)	0.2 (0.2-0.3)	0.4 (0.3-0.4)
American Indian	110	3 (3.0)	7.6 (6.2-9.2)	3.6 (2.7-4.7)	2.7 (1.8-3.7)	0.8 (0.4-1.5)	0.1 (0.0-0.4)
Asian/Pacific Islander							
Overall^c^	1845	1696 (91.9)	5.5 (5.3-5.8)	1.5 (1.3-1.6)	1.8 (1.6-1.9)	0.1 (0.0-0.1)	1.8 (1.6-1.9)
Chinese	467	442 (94.6)	5.0 (4.6-5.5)	0.7 (0.6-0.9)	1.2 (1.0-1.5)	NA	2.6 (2.3-3.0)
Filipino	375	355 (94.7)	4.4 (4.0-4.9)	0.6 (0.4-0.8)	2.5 (2.2-2.9)	NA	0.8 (0.6-1.0)
Japanese	146	104 (71.2)	3.9 (3.3-4.7)	1.8 (1.4-2.3)	1.6 (1.2-2.1)	NA	0.2 (0.1-0.5)
Korean	196	195 (99.5)	7.0 (6.0-8.1)	1.9 (1.4-2.6)	1.8 (1.3-2.3)	NA	2.8 (2.2-3.5)
Pacific Islander	59	24 (40.7)	7.2 (5.4-9.3)	2.3 (1.4-3.5)	2.9 (1.8-4.6)	NA	1.2 (0.6-2.1)
South Asian^d^	58	55 (94.8)	2.4 (1.8-3.2)	1.0 (0.7-1.5)	1.1 (0.7-1.7)	NA	0.2 (0.0-0.4)
Vietnamese	354	351 (99.2)	11.1 (10.0-12.4)	4.8 (4.0-5.7)	2.1 (1.6-2.7)	NA	3.5 (2.9-4.2)
Other Southeastern Asian							
Overall^c^	123	121 (98.4)	9.9 (8.1-11.9)	2.9 (2.0-4.0)	2.5 (1.6-3.7)	NA	3.6 (2.6-4.8)
Cambodian	60	60 (100)	14.0 (10.6-18.2)	6.3 (4.1-9.2)	NA	NA	3.4 (1.9-5.6)
Hmong	13	12 (92.3)	NA	NA	NA	NA	NA
Laotian	24	24 (100)	9.6 (6.0-14.6)	NA	NA	NA	5.4 (2.7-9.5)
Thai	26	25 (96.2)	7.2 (4.4-11.5)	NA	NA	NA	2.7 (1.2-5.8)
Latino							
Overall^c^	2724	1592 (58.4)	6.4 (6.1-6.6)	2.4 (2.2-2.5)	2.9 (2.7-3.1)	0.5 (0.4-0.5)	0.1 (0.1-0.1)
US-born	1132	0	7.4 (6.9-7.8)	3.5 (3.2-3.8)	2.9 (2.6-3.2)	0.5 (0.4-0.6)	0.1 (0.0-0.1)
Born elsewhere	1592	1592 (100)	6.0 (5.7-6.3)	1.8 (1.7-2.0)	3.0 (2.8-3.2)	0.5 (0.4-0.6)	0.1 (0.1-0.2)
Non-Latino Black	603	44 (7.3)	4.7 (4.3-5.1)	3.0 (2.7-3.3)	1.1 (0.9-1.3)	0.3 (0.2-0.4)	0.1 (0.0-0.2)
Non-Latino White	2809	8670 (13.2)	2.6 (2.5-2.7)	1.1 (1.1-1.2)	0.8 (0.8-0.9)	0.2 (0.2-0.3)	0.0 (0.0-0.1)

Abbreviations: AAIR, age-adjusted incidence rate (for 2000 US standard population); ALD, alcoholic liver disease; HBV, hepatitis B virus; HCC, hepatocellular carcinoma; HCV, hepatitis C virus; MASLD, metabolic dysfunction–associated steatotic liver disease; NA, not available (small numbers of observations are not shown due to confidentiality protection).

^a^ Includes all listed as well as cryptogenic and others (eg, genetic, autoimmune).

^b^ All races and ethnicities combined only includes those listed (excludes multiracial, other, and unknown race).

^c^ Includes all patients of this race and ethnicity, not just listed groups.

^d^ Includes all patients of races related to Bangladesh, India, Nepal, Pakistan, and Sri Lanka.

**Figure 1.**
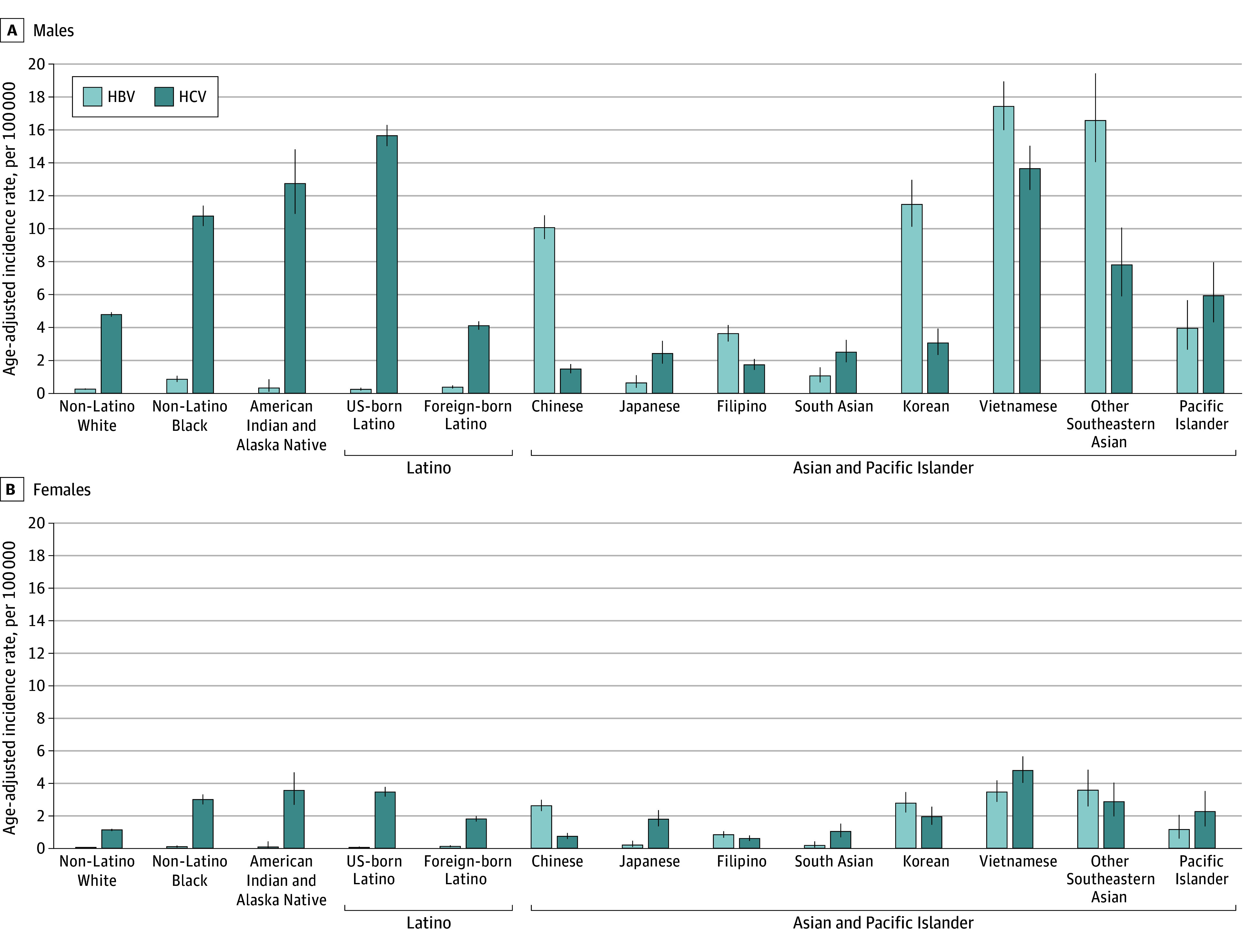
Age-Adjusted Incidence Rates for Viral Etiology Hepatocellular Carcinoma by Sex and Race and Ethnicity HBV indicates hepatitis B virus; HCV, hepatitis C virus.

AAIRs varied substantially by etiology. HCV-related HCC was the most common cause in all Black, US-born Latino, and White populations; Latino populations born outside the US predominantly had ALD-related HCC men and MASLD-related HCC in women. In contrast, HBV-related HCC was the predominant cause of HCC among Asian and Pacific Islander individuals when considered as a single category. MASLD-related HCC disproportionately affected American Indian (4.2 per 100 000 in men, 2.7 per 100 000 in women), Asian and Pacific Islander (3.9 per 100 000 in men; 1.8 per 100 000 in women), and Latino (4.4 per 100 000 in men; 2.9 per 100 000 in women) populations. HBV-related HCC remained important in some Asian and Pacific Islander subgroups (Cambodian [18.3 per 100 000 in men; 3.4 per 100 000 in women], Chinese [10.1 per 100 000 in men; 2.6 per 100 000 in women], Korean [11.5 per 100 000 in men; 2.8 per 100 000 in women], Laotian (25.0 per 100 000 in men; 5.4 per 100 000 in women) and Vietnamese (16.6 per 100 000 in men; 3.5 per 100 000 in women]), but not all subgroups, and HBV-related HCC declined overall. Figure 1 details viral etiology–specific patterns across select Asian and Pacific Islander subgroups, showing high rates of HBV-related HCC and HCV-related HCC in Vietnamese and other Southeastern Asian populations. HCV-related HCC rates were highest among American Indian (12.8 per 100 000 in men; 3.6 per 100 000 in women), Black (10.8 per 100 000 in men; 3.0 per 100 000 in women), and US-born Latino (15.7 per 100 000 in men; 3.5 per 100 000 in women) populations and specific Asian groups (particularly Cambodian [15.5 per 100 000 in men; 6.3 per 100 000 in women) and Vietnamese [13.7 per 100 000 in men; 4.8 per 100 000 in women]). eFigure in Supplement 1 illustrates age-specific HCV-related HCC rates, highlighting the 1945 to 1965 birth cohort effect in US-born populations but not in Asian and Pacific Islander populations or Latino people born outside the US.

ALD-related HCC was most prevalent among American Indian and US-born Latino males but less so among Asian and Pacific Islander, Black, and White males. MASLD-related HCC rates were highest among American Indian, Asian and Pacific Islander (particularly Filipino and other Southeast Asian), and Latino populations. Sex ratios by etiology varied, with ALD-related HCC showing the largest male-to-female ratio (9.1 to 1).

### Trends in HCC Incidence by Etiology

HCC trends for all races and ethnicities combined increased from 2010 to 2014 but reversed in 2014, with overall rates changing by −3.1% (95% CI −4.8% to −1.4%; *P* = .001) in men and −3.2% (95% CI, −10.9% to −0.9%; *P* = .02) in women annually (Table 3). The steepest declines occurred among Asian and Pacific Islander (2010 to 2018) and White (2014 to 2018) populations. However, declines were not observed in Black males, US-born Latino females, and Latino people born outside the US of both sexes (eTable 5 in Supplement 1).

**Table 3.** HCC Trends by Etiology, Race and Ethnicity, and Sex

Race and ethnicity	Estimate (95% CI)				
	ALL HCC	HCV-HCC	MASLD-HCC	ALD-HCC	HBV-HCC
**Males**					
Males combined^a^					
AAIR, per 100 000 population					
2010 to 2011	13.3 (12.9 to 13.7)	6.1 (5.9 to 6.4)	2.8 (2.6 to 3.0)	1.8 (1.7 to 1.9)	1.6 (1.5 to 1.7)
2017 to 2018	12.3 (12.0 to 12.6)	5.1 (4.9 to 5.3)	3.1 (2.9 to 3.3)	2.0 (1.9 to 2.1)	1.3 (1.2 to 1.4)
APC, %					
2010 to 2014	1.0 (−0.8 to 2.8)	1.4 (-1.9 to 4.8)	1.9 (0.8 to 3.0)	1.9 (0.6 to 3.1)	−3.0 (−4.9 to −1.1)
2014 to 2018	−3.1 (−4.8 to −1.4)	−6.7 (-9.8 to −3.5)			
Asian and Pacific Islander					
AAIR, per 100 000 population					
2010 to 2011	20.2 (18.9 to 21.5)	4.6 (4.0 to 5.3)	4.3 (3.7 to 5.0)	0.9 (0.7 to 1.2)	8.9 (8.1 to 9.7)
2017 to 2018	14.6 (13.7 to 15.6)	2.7 (2.3 to 3.2)	3.6 (3.1 to 4.1)	6.5 (5.9 to 7.1)	6.5 (5.9 to 7.1)
APC: 2010 to 2018, %	−4.1 (−5.7 to −2.5)	−7.1 (−8.8 to −5.4)	−2.2 (−4.3 to −0.1)	0.0 (−6.2 to 6.5)	−4.0 (−6.0 to −1.9)
Latino					
AAIR, per 100 000 population					
2010 to 2011	18.9 (17.p to 19.9)	8.9 (8.3 to 9.5)	4.5 (4.0 to 5.1)	3.9 (3.4 to 4.4)	0.4 (0.3 to 0.6)
2017 to 2018	17.9 (16.9 to 18.6)	6.7 (6.2 to 7.2)	5.0 (4.5 to 5.5)	4.7 (4.3 to 5.2)	0.3 (0.2 to 0.4)
APC, %					
2010 to 2015	−0.9 (−1.6 to −0.1)	−0.6 (−3.5 to 2.4)	1.3 (−2.0 to 4.7)	2.3 (0.0 to 4.6)	−3.2 (−10.3 to 4.5)
2015 to 2018		−9.3 (−15.3 to −2.9)			
Non-Latino Black					
AAIR, per 100 000 population					
2010 to 2011	16.3 (14.6 to 18.1)	10.8 (9.5 to 12.2)	2.3 (1.6 to 3.2)	1.1 (0.7 to 1.7)	1.1 (0.7 to 1.6)
2017 to 2018	15.2 (13.7 to 16.9)	9.7 (8.5 to 11.0)	2.5 (1.8 to 3.3)	1.6 (1.1 to 2.2)	0.7 (0.4 to 1.1)
APC: 2010 to 2018, %	−1.3 (−4.2 to 1.6)	−1.8 (−5.9 to 2.4)	2.4 (−4.5 to 9.8)	4.4 (−4.3 to 13.9)	−9.0 (−16.3 to −1.0)
Non-Latino White					
AAIR, per 100 000 population					
2010 to 2011	9.3 (8.9 to 9.7)	4.9 (4.6 to 5.2)	2.0 (1.8 to 2.2)	1.3 (1.1 to 1.5)	0.3 (0.2 to 0.4)
2017 to 2018	8.8 (8.4 to 9.2)	4.3 (4.0 to 4.6)	2.4 (2.2 to 2.6)	1.3 (1.2 to 1.5)	0.2 (0.1 to 0.3)
APC, %					
2010 to 2013	2.4 (−1.2 to 6.3)	4.5 (−5.6 to 15.5)	2.9 (1.3 to 4.5)	−0.4 (−1.9 to 1.1)	−6.0 (−9.7 to −2.0)
2013 to 2018	−2.8 (−4.3 to −1.2)	−5.2 (−9.3 to −0.9)			
**Females**					
Females combined^a^					
AAIR, per 100 000 population					
2010 to 2011	4.0 (3.8 to 4.2)	1.7 (1.6 to 1.8)	1.3 (1.2 to 1.4)	0.2 (0.2 to 0.2)	0.4 (0.3 to 0.5)
2017 to 2018	3.9 (3.7 to 4.1)	1.4 (1.3 to 1.5)	1.6 (1.5 to 1.7)	0.3 (0.3 to 0.4)	0.3 (0.3 to 0.4)
APC, %					
2010 to 2014	2.9 (0.4 to 12.3)	1.6 (−1.8 to 13.8)	3.2 (1.1 to 5.7)	3.8 (0.2 to 8.2)	−3.2 (−5.5 to −0.8)
2014 to 2018	−3.2 (−10.9 to −0.9)	−6.9 (−16.8 to −3.8)			
Asian/Pacific Islander					
AAIR, per 100 000 population					
2010 to 2011	6.7 (6.1 to 7.4)	2.1 (1.7 to 2.5)	1.8 (1.5 to 2.2)	0.1 (0.0 to 0.2)	2.0 (1.7 to 2.4)
2017 to 2018	4.4 (3.9 to 4.9)	1.0 (0.8 to 1.3)	1.6 (1.3 to 1.9)	0.1 (0.1 to 0.2)	1.5 (1.3 to 1.8)
APC: 2010 to 2018, %	−5.5 (−7.7 to −3.3)	−11.0 (−14.8 to −7.4)	−0.4 (−8.3 to 9.0)	−8.4 (−32.0 to 21.8)	−5.2 (−8.3 to −2.1)
Latino					
AAIR, per 100 000 population					
2010 to 2011	6.1 (5.6 to 6.7)	2.3 (2.0 to 2.6)	2.7 (2.3 to 3.3)	0.4 (0.3 to 0.6)	0.1 (0.1 to 0.2)
2017 to 2018	6.8 (6.3 to 7.3)	2.2 (1.9 to 2.5)	3.4 (3.1 to 3.8)	0.5 (0.4 to 0.7)	0.1 (0.1 to 0.2)
APC, %					
2010 to 2013	1.1 (−1.5 to 4.0)	8.2 (−6.9 to 47.7)	3.1 (−1.8 to 9.1)	3.1 (−5.0 to 13.1)	1.1 (−1.5 to 4.0)
2013 to 2018		−5.2 (−27.5 to 6.8)			
Non-Latino Black					
AAIR, per 100 000 population					
2010 to 2011	5.4 (4.5 to 6.4)	3.5 (2.8 to 4.3)	0.9 (0.6 to 1.4)	0.3 (0.1 to 0.6)	0.2 (0.1 to 0.5)
2017 to 2018	4.1 (3.4 to 4.9)	2.7 (2.2 to 3.3)	1.1 (0.7 to 1.6)	0.2 (0.1 to 0.4)	0.0 (0.0 to 0.2)
APC: 2010 to 2018, %	−2.4 (−4.7 to 0.0)	−3.1 (−7.3 to 1.2)	3.1 (−1.9 to 8.4)	−0.8 (−24.9 to 31.6)	−18.8 (−44.3 to 5.8)
Non-Latino White					
AAIR, per 100 000 population					
2010 to 2011	2.4 (2.2 to 2.6)	1.1 (1.0 to 1.3)	0.7 (0.6 to 0.8)	0.2 (0.2 to 0.3)	0.1 (0.1 to 0.1)
2017 to 2018	2.4 (2.2 to 2.6)	1.0 (0.9 to 1.1)	0.9 (0.8 to 1.0)	0.2 (0.1 to 0.3)	0.1 (0.1 to 0.1)
APC, %					
2010 to 2014	5.5 (2.0 to 16.6)	4.1 (0.5 to 13.0)	2.9 (−0.1 to 6.1)	6.7 (−3.3 to 17.9)	1.9 (−7.4 to 13.1)
2014 to 2018	−4.1 (−12.6 to −0.8)	−6.8 (−14.0 to −3.3)			

Abbreviations: AAIR, age-adjusted incidence rate; ALD, alcoholic liver disease; APC, annual percentage change; HBV, hepatitis B virus; HCC, hepatocellular carcinoma; HCV, hepatitis C virus; MASLD, metabolic dysfunction–associated steatotic liver disease.

^a^ All races and ethnicities combined only includes those listed here (excludes multiracial, other, and unknown race).

**Figure 2.**
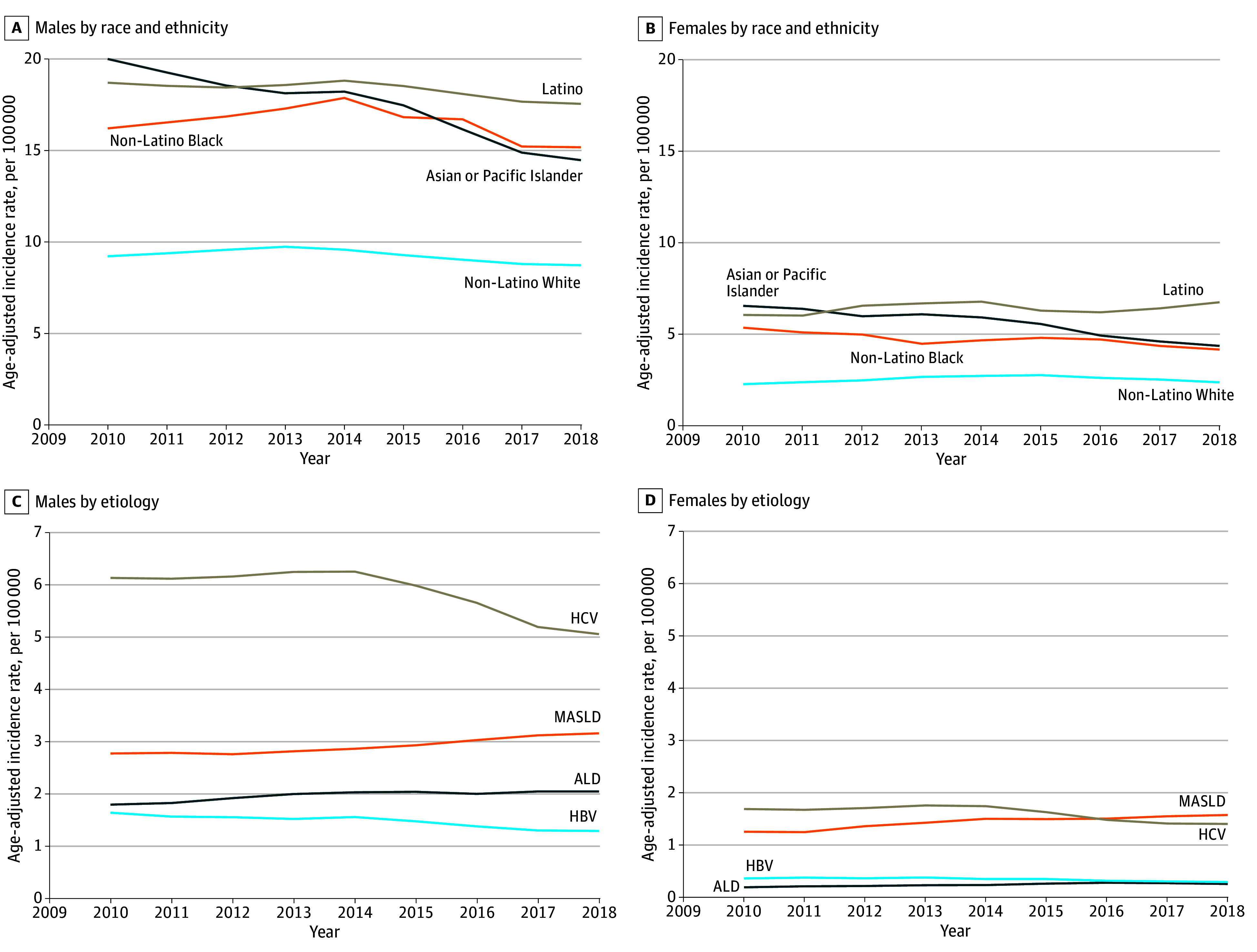
Three-Year Moving Mean Age-Adjusted Incidence Rates for Hepatocellular Carcinoma ALD indicates alcoholic liver disease; HBV, hepatitis B virus; HCV, hepatitis C virus; MASLD, metabolic dysfunction–associated live disease.

By etiology, HCV-related HCC rates changed by −7.0% (95% CI, −8.9% to −5.4%; *P* = .001) annually after 2014, with the fastest declines among Asian and Pacific Islander populations, while changes among Black populations were not statistically significant (Table 3). HBV-related HCC rates changed by −3.0% (95% CI, −4.9% to −1.1%; *P* = .02) annually during 2010 to 2018. Conversely, MASLD-related HCC and ALD-related HCC rates increased (MASLD: 1.9%; 95% CI, 0.8% to 3.0%); ALD: 1.9%; 95% CI, 0.6% to 3.1%) (Figure 2), making MASLD-related HCC the leading cause of HCC in women by 2017 to 2018. eTable 6 in Supplement 1 highlights age-specific trends, showing that while HCV-related HCC and HBV-related HCC decreased, MASLD-related HCC and ALD-related HCC significantly increased, particularly in middle-aged and older adults, contributing to a substantial increase in the median (IQR) age of HCC diagnosis from 63 (16) years in 2010 to 67 (14) years in 2018. Sensitivity analyses for imputed variables, including extreme scenarios, showed no notable variation from the reported results.

## Discussion

This cohort study describes important trends and disparities in HCC across racial and ethnic groups in California, associated with a complex interplay of socioeconomic, cultural, behavioral, and genetic factors. HCC risk varies up to 6-fold among populations, emphasizing the need for targeted public health interventions that go beyond generic cancer prevention and treatment strategies. While viral etiologies, especially HCV and HBV, play a major role in driving disparities in HCC incidence, there is also considerable heterogeneity in ALD-related HCC and MASLD-related HCC.

Our findings confirm that HCV remains the leading cause of HCC among predominantly US-born populations, including American Indian, Black, Latino, and White populations. High rates for HCV-related HCC are largely attributable to the iatrogenic spread of the virus from 1960 to 1992, the sharing of infected needles, and unsterilized tattoo practices, among other factors.^44^ HCV-related HCC is most prevalent among US-born American Indian, Black, and Latino men. These disparities highlight inequities in the social determinants of HCV^45^ and possibly limited access to timely DAA therapy.^46,47^ Consistent with previous studies,^8^ we found the highest rates of HCV-related HCC among US-born Latino men, who have 3 times higher rates than Latinos born outside the US, who often originate from regions with lower HCV prevalence.^48^

There was a strong association of race and ethnicity with etiology of HCC. ALD-related HCC rates were highest among American Indian and US-born Latino populations, who also experienced elevated rates of HCV-related HCC and MASLD-related HCC. This pattern differs from that among Black individuals, who predominantly face high rates of HCV-related HCC. These findings underscore the shared social and health care inequities among non-Black minoritized racial and ethnic groups, such as American Indian, Asian and Pacific Islander, and Latino populations, emphasizing the need to address social determinants of health that drive these disparities through distinct pathways. HBV, the other major cause of HCC, remains rare in California for people who are not Asian or Pacific Islander.

A novel contribution of our study is its granular analysis of Asian and Pacific Islander populations, often treated as a monolithic entity in health research. We found that while HBV was the leading cause of HCC among Cambodian, Chinese, Hmong, Korean, Laotian, and Vietnamese populations, it was rare among Japanese and South Asian populations, whose HCC rates aligned with those of White populations. For example, Vietnamese males experienced HBV-related HCC rates 30 times higher than Japanese males. This stark disparity likely reflects differences in baseline HBV prevalence, although inequities in testing for chronic HBV infection, referral, and treatment access across Asian subgroups cannot be ruled out.^49^

Interestingly, this heterogeneity extends to HCV-related HCC, with particularly high rates observed among Cambodian and Vietnamese populations, reflecting elevated HCV prevalence in their countries of origin.^50^ These findings challenge the typical focus on HBV prevention for Asian and Pacific Islander populations and advocate for broader screening efforts that include both HBV and HCV, in line with Centers for Disease Control and Prevention recommendations.^26,29^

Another key finding is the prominence of MASLD as a leading cause of HCC among Filipino, Pacific Islander, and South Asian people, with MASLD-related HCC rates higher across all Asian subgroups compared with White people. Research shows that the prevalence of MASLD in Asian populations abroad exceeds that in North American populations.^51^ However, the burden of MASLD-related HCC extends beyond Asian groups; it is increasingly driving HCC incidence across Latino and other racial-ethnic groups in the US. Increasing obesity rates, now affecting more than 42% of US adults, combined with the increasing prevalence of diabetes and metabolic syndrome, are fueling the upward trend in MASLD as a source of HCC.^52,53^ Further research into MASLD within these diverse populations is warranted, particularly given the variations in obesity, diabetes, and metabolic syndrome prevalence.^54,55,56^

Migration histories, socioeconomic status, cultural factors, and differing perceptions of alcohol use across these populations may help explain the root causes of all 4 major precursors of HCC incidence and find better targets for intervention. Unfortunately, the continued reliance on broad racial and ethnic categories, such as Asian combined with Pacific Islander, Black, and Latino, hinders progress. More granular data and disaggregation are essential to address the specific needs of these populations and improve health outcomes.

Temporal trends in this study highlight the evolving landscape of HCC etiology. The overall favorable trend is largely driven by reductions in HCV-related HCC, particularly in historically high-incidence populations, reflecting the impact of HCV screening and DAAs. Among Asian and Pacific Islander populations, declines in HBV-related HCC align with the success of vaccination programs in Asia,^57^ increased cultural awareness,^58^ and improved screening and vaccination efforts in the US.^41,59,60,61^ These trends have significantly reduced HCC rates in Asian and Pacific Islander populations, bringing them in line with those observed in Black and Latino populations. However, MASLD- and ALD-related HCC are on the rise across the population as a whole, posing new public health challenges.

Regarding the decline of HCV-related HCC, a significant decrease was evident among Asian and Pacific Islander people, US-born Latino males, White people, and, to a less extent, Black people. However, the patterns among Black, US-born Latino, and White populations are distinct from those of the Asian and Pacific Islander population. Among US-born populations, the decline appears closely tied to the advent of DAAs. In contrast, the decline in HCV-related HCC among Asian and Pacific Islander people predates DAA availability. Moreover, Asian and Pacific Islander people do not exhibit the high-risk profile linked to the 1945 to 1965 US birth cohort, and these populations show unique HCV genotypes, such as 1b in Chinese people and 6a in Southeast Asian people,^62,63^ contrasting with the predominant 1a genotype in US-born populations, reinforcing the different epidemiology of HCV-related HCC among Asian and Pacific Islander people. For actionable disparities in California, the recognition of some Asian and Pacific Islander groups with very high incidence of viral hepatitis-related HCC (eg, Cambodian and Vietnamese), slower HCV-related HCC rate reductions among Black males, and persistently higher HCC rates among non-White populations compared with their counterparts in Florida^8^ highlight opportunities for improving prevention.

### Strengths and Limitations

This study’s main strength lies in its use of individual-level data for California’s entire HCC population, offering a nuanced examination of etiology among Asian and Pacific Islander subgroups. This study also has some limitations. A main limitation is the pre–COVID-19 timeframe, which limits applicability to recent trends. Additionally, interpretation of AAIRs for smaller Asian and Pacific Islander groups (eg, Cambodian, Hmong, Laotian, Thai) should be cautious due to low numbers. The leading causes of HCC identified in this study reflect patterns specific to California and may differ in other populations. However, we observed notable similarities in rates and proportions among subgroups born outside the US (race-ethnicity level II) across states, likely influenced by shared migration histories and limited acculturation to local environments. Furthermore, while our post-DAA trend analysis reveals critical shifts in HCV-related HCC trends, the limited period of DAA availability may not entirely explain the observed shift in the epidemiological trajectory of a complex disease like HCV-related HCC.

## Conclusions

The findings of this retrospective cohort study underscore the critical need for tailored public health strategies to address HCC’s varied etiologies. The persisting high rates of HCV-related HCC and HBV-related HCC suggest that screening for both HCV and HBV remains suboptimal, with some populations lacking timely access to viral hepatitis treatments. Improving implementation of HBV and HCV screening is particularly important in areas where higher-risk populations reside. Addressing the low prevalence of viral hepatitis screening requires a concerted effort involving medical practitioners, including enhanced awareness, training, and adherence to screening guidelines. Equally important is ensuring effective linkage to care for patients diagnosed with chronic HBV or HCV, enabling timely initiation of treatment. Patient-centered programs, including language-concordant education and outreach, culturally appropriate patient navigators, and stigma reduction efforts, are vital to improve both screening uptake and care continuity. These tailored strategies are essential to advancing health equity and mitigating the burden of HCC in diverse and underserved populations.

As MASLD emerged as a leading HCC driver, particularly among women since 2017 to 2018, lifestyle and metabolic health interventions will also be critical in high-risk groups. Reducing HCC incidence and improving outcomes will require a combination of granular data collection and analysis, targeted interventions, and equity-focused health care policies.

## Notes

Supplement 1. **eFigure.** Age-Specific Incidence Rate for HCV-HCC With Birth Cohort Information **eTable 1.** Diagnostic Code Definitions for HCC California Data, 2010-2018 **eTable 2.** Patient Region of Residence Defined by County of Residence at the Time of HCC Diagnosis **eTable 3.** Stage at Diagnosis by Detailed Race/Ethnicity of HCC Cases **eTable 4.** Insurance Type by Detailed Race/Ethnicity of HCC Cases **eTable 5.** Age-Adjusted Incidence Rates per 100,000 and Temporal Trends Annual Percent Change for HCC by Detailed Race/Ethnicity and Sex **eTable 6.** Temporal Trends Annual Percent Change for HCC by Age Group and Etiology

Supplement 2.
Data Sharing Statement

## References

[1] American Cancer Society. Cancer facts & figures 2024. Accessed January 9, 2025. https://www.cancer.org/research/cancer-facts-statistics/all-cancer-facts-figures/2024-cancer-facts-figures.html

[2] Jemal A, Ward EM, Johnson CJ, . Annual report to the nation on the status of cancer, 1975-2014, featuring survival. J Natl Cancer Inst. 2017;109(9):djx030. doi:10.1093/jnci/djx030 28376154 PMC5409140

[3] Rich NE, Hester C, Odewole M, . Racial and ethnic differences in presentation and outcomes of hepatocellular carcinoma. Clin Gastroenterol Hepatol. 2019;17(3):551-559.e1. doi:10.1016/j.cgh.2018.05.039 29859983 PMC6274621

[4] Barzi A, Zhou K, Wang S, Dodge JL, El-Khoueiry A, Setiawan VW. Etiology and outcomes of hepatocellular carcinoma in an ethnically diverse population: the multiethnic cohort. Cancers (Basel). 2021;13(14):3476. doi:10.3390/cancers13143476 34298690 PMC8305188

[5] Cranford HM, Jones PD, Wong RJ, . Hepatocellular carcinoma etiology drives survival outcomes: a population-based analysis. Cancer Epidemiol Biomarkers Prev. 2024;33(12):1717-1726. doi:10.1158/1055-9965.EPI-24-0626 39240221 PMC11611647

[6] National Cancer Institute. Surveillance, Epidemiology, and End Results Program incidence data, 1975-2021. Accessed January 9, 2025. http://www.seer.cancer.gov

[7] Islami F, Miller KD, Siegel RL, Fedewa SA, Ward EM, Jemal A. Disparities in liver cancer occurrence in the United States by race/ethnicity and state. CA Cancer J Clin. 2017;67(4):273-289. doi:10.3322/caac.21402 28586094

[8] Pinheiro PS, Jones PD, Medina H, . Incidence of etiology-specific hepatocellular carcinoma: diverging trends and significant heterogeneity by race and ethnicity. Clin Gastroenterol Hepatol. 2024;22(3):562-571.e8. doi:10.1016/j.cgh.2023.08.016 37678486 PMC10915102

[9] Miller BA, Chu KC, Hankey BF, Ries LA. Cancer incidence and mortality patterns among specific Asian and Pacific Islander populations in the U.S. Cancer Causes Control. 2008;19(3):227-256. doi:10.1007/s10552-007-9088-3 18066673 PMC2268721

[10] Dee EC. Disaggregated cancer research and intervention for Asian American and Pacific Islander populations. JAMA Netw Open. 2024;7(11):e2442419. doi:10.1001/jamanetworkopen.2024.42419 39495517

[11] Medina HN, Callahan KE, Morris CR, Thompson CA, Siweya A, Pinheiro PS. Cancer Mortality Disparities among Asian American and Native Hawaiian/Pacific Islander Populations in California. Cancer Epidemiol Biomarkers Prev. 2021;30(7):1387-1396. doi:10.1158/1055-9965.EPI-20-1528 33879454 PMC8254771

[12] Jin H, Pinheiro PS, Xu J, Amei A. Cancer incidence among Asian American populations in the United States, 2009-2011. Int J Cancer. 2016;138(9):2136-2145. doi:10.1002/ijc.29958 26661680 PMC5283572

[13] Pollack HJ, Kwon SC, Wang SH, Wyatt LC, Trinh-Shevrin C; AAHBP Coalition. Chronic hepatitis B and liver cancer risks among Asian immigrants in New York City: results from a large, community-based screening, evaluation, and treatment program. Cancer Epidemiol Biomarkers Prev. 2014;23(11):2229-2239. doi:10.1158/1055-9965.EPI-14-0491 25368398 PMC4373070

[14] Kaliszewski M. Alcohol and drug use among Asian Americans. *American Addiction Centers*. November 21, 2024. Accessed January 9, 2025. https://americanaddictioncenters.org/rehab-guide/addiction-statistics-demographics/asian-americans

[15] Truong E, Yeo YH, Cook-Wiens G, . Nonalcoholic fatty liver disease prevalence and severity in Asian Americans from the national health and nutrition examination surveys 2017-2018. Hepatol Commun. 2022;6(9):2253-2261. doi:10.1002/hep4.1981 35527706 PMC9426392

[16] AASLD/IDSA HCV Guidance Panel. Hepatitis C guidance: AASLD-IDSA recommendations for testing, managing, and treating adults infected with hepatitis C virus. Hepatology. 2015;62(3):932-954. doi:10.1002/hep.27950 26111063

[17] Moyer VA; U.S. Preventive Services Task Force. Screening for hepatitis C virus infection in adults: U.S. Preventive Services Task Force recommendation statement. Ann Intern Med. 2013;159(5):349-357. doi:10.7326/0003-4819-159-5-201309030-00672 23798026

[18] Ryerson AB, Eheman CR, Altekruse SF, . Annual report to the nation on the status of cancer, 1975-2012, featuring the increasing incidence of liver cancer. Cancer. 2016;122(9):1312-1337. doi:10.1002/cncr.29936 26959385 PMC4840031

[19] Pinheiro PS, Callahan KE, Boscoe FP, . Cancer site-specific disparities in New York, including the 1945-1965 birth cohort’s impact on liver cancer patterns. Cancer Epidemiol Biomarkers Prev. 2018;27(8):917-927. doi:10.1158/1055-9965.EPI-18-0194 30026296 PMC6193556

[20] Torre LA, Sauer AM, Chen MS Jr, Kagawa-Singer M, Jemal A, Siegel RL. Cancer statistics for Asian Americans, Native Hawaiians, and Pacific Islanders, 2016: converging incidence in males and females. CA Cancer J Clin. 2016;66(3):182-202. doi:10.3322/caac.21335 26766789 PMC5325676

[21] California Viral Hepatitis Coordinating Committee and California Department of Public Health. California viral hepatitis prevention strategic plan, 2016-2020. Accessed January 9, 2025. https://www.globalhep.org/tools-resources/action-plans/california-viral-hepatitis-prevention-strategic-plan-2016-2020-report

[22] Centers for Disease Control and Prevention. Behavioral Risk Factor Surveillance System annual survey data. Accessed January 9, 2025. https://www.cdc.gov/brfss/annual_data/annual_data.htm

[23] Kanwal F, Kramer J, Asch SM, Chayanupatkul M, Cao Y, El-Serag HB. Risk of hepatocellular cancer in HCV patients treated with direct-acting antiviral agents. Gastroenterology. 2017;153(4):996-1005.e1. doi:10.1053/j.gastro.2017.06.012 28642197

[24] Lockart I, Yeo MGH, Hajarizadeh B, Dore GJ, Danta M. HCC incidence after hepatitis C cure among patients with advanced fibrosis or cirrhosis: a meta-analysis. Hepatology. 2022;76(1):139-154. doi:10.1002/hep.32341 35030279 PMC9303770

[25] Rich NE, Yopp AC, Singal AG, Murphy CC. Hepatocellular carcinoma incidence is decreasing among younger adults in the United States. Clin Gastroenterol Hepatol. 2020;18(1):242-248.e5. doi:10.1016/j.cgh.2019.04.043 31042582 PMC6817412

[26] Conners EE, Panagiotakopoulos L, Hofmeister MG, . Screening and testing for hepatitis B virus infection: CDC recommendations—United States, 2023. MMWR Recomm Rep. 2023;72(1):1-25. doi:10.15585/mmwr.rr7201a1 36893044 PMC9997714

[27] Gnanapandithan K, Ghali MP. Self-awareness of hepatitis C infection in the United States: a cross-sectional study based on the National Health Nutrition and Examination Survey. PLoS One. 2023;18(10):e0293315. doi:10.1371/journal.pone.0293315 37874815 PMC10597475

[28] Roberts H, Ly KN, Yin S, Hughes E, Teshale E, Jiles R. Prevalence of HBV infection, vaccine-induced immunity, and susceptibility among at-risk populations: US households, 2013-2018. Hepatology. 2021;74(5):2353-2365. doi:10.1002/hep.31991 34097776

[29] Schillie S, Wester C, Osborne M, Wesolowski L, Ryerson AB. CDC recommendations for hepatitis C screening among adults—United States, 2020. MMWR Recomm Rep. 2020;69(2):1-17. doi:10.15585/mmwr.rr6902a1 32271723 PMC7147910

[30] Zhou K, Terrault NA. Gaps in viral hepatitis awareness in the United States in a population-based study. Clin Gastroenterol Hepatol. 2020;18(1):188-195.e4. doi:10.1016/j.cgh.2019.05.047 31173892 PMC8028744

[31] Fleurence RL, Collins FS. A national hepatitis C elimination program in the United States: a historic opportunity. JAMA. 2023;329(15):1251-1252. doi:10.1001/jama.2023.3692 36892976

[32] North American Association of Central Cancer Registries. NAACCR certification by registry and year, certified registries. Accessed September 4, 2024. https://www.naaccr.org/certified-registries/

[33] Makarova-Rusher OV, Altekruse SF, McNeel TS, . Population attributable fractions of risk factors for hepatocellular carcinoma in the United States. Cancer. 2016;122(11):1757-1765. doi:10.1002/cncr.29971 26998818 PMC5548177

[34] Welzel TM, Graubard BI, Quraishi S, . Population-attributable fractions of risk factors for hepatocellular carcinoma in the United States. Am J Gastroenterol. 2013;108(8):1314-1321. doi:10.1038/ajg.2013.160 23752878 PMC4105976

[35] Karim MA, Singal AG, Kum HC, . Clinical characteristics and outcomes of nonalcoholic fatty liver disease-associated hepatocellular carcinoma in the United States. Clin Gastroenterol Hepatol. 2023;21(3):670-680.e18. doi:10.1016/j.cgh.2022.03.010 35307595 PMC9481743

[36] Pinheiro PS, Medina HN, Callahan KE, . The association between etiology of hepatocellular carcinoma and race-ethnicity in Florida. Liver Int. 2020;40(5):1201-1210. doi:10.1111/liv.14409 32087002 PMC8637930

[37] Beste LA, Leipertz SL, Green PK, Dominitz JA, Ross D, Ioannou GN. Trends in burden of cirrhosis and hepatocellular carcinoma by underlying liver disease in US veterans, 2001-2013. Gastroenterology. 2015;149(6):1471-1482.e5. doi:10.1053/j.gastro.2015.07.056 26255044

[38] California Cancer Registry. CCR data dictionary May 2024. Accessed January 10, 2025. https://www.ccrcal.org/retrieve-data/data-library/#273-291-data-dictionary-completeness-tables

[39] U.S. Census Bureau. American Community Survey 5-year estimates: comparison profiles 5-year. Accessed January 10, 2025. https://catalog.data.gov/dataset/american-community-survey-5-year-estimates-comparison-profiles-5-year

[40] North American Association of Central Cancer Registries. NAACCR Hispanic and Asian/Pacific Islander identification algorithm [NHAPIIA v19]. Accessed January 10, 2025. https://www.naaccr.org/nhapiia_2017_04_21-v17-0/

[41] Sangaramoorthy M, Yang J, DeRouen MC, . Disparities in hepatocellular carcinoma incidence in California: an update. Cancer Epidemiol Biomarkers Prev. 2020;29(1):79-87. doi:10.1158/1055-9965.EPI-19-0560 31719066 PMC6986425

[42] Ruggles S, Flood S, Sobek M, . IPUMS USA: version 15.0 [dataset]. Accessed January 10, 2025. https://www.ipums.org/projects/ipums-usa/d010.V15.0

[43] National Cancer Institute. Joinpoint Regression Program: version 4.9.1.0—April 2022. Accessed January 10, 2025. https://surveillance.cancer.gov/joinpoint/

[44] Smith BD, Morgan RL, Beckett GA, ; Centers for Disease Control and Prevention. Recommendations for the identification of chronic hepatitis C virus infection among persons born during 1945-1965. MMWR Recomm Rep. 2012;61(RR-4):1-32. 22895429

[45] Kuniholm MH, Jung M, Everhart JE, . Prevalence of hepatitis C virus infection in US Hispanic/Latino adults: results from the NHANES 2007-2010 and HCHS/SOL studies. J Infect Dis. 2014;209(10):1585-1590. doi:10.1093/infdis/jit672 24423693 PMC3997577

[46] Kapadia SN, Zhang H, Gonzalez CJ, . Hepatitis C treatment initiation among US Medicaid enrollees. JAMA Netw Open. 2023;6(8):e2327326. doi:10.1001/jamanetworkopen.2023.27326 37540513 PMC10403776

[47] Wong RJ, Jain MK, Therapondos G, . Race/ethnicity and insurance status disparities in access to direct acting antivirals for hepatitis C virus treatment. Am J Gastroenterol. 2018;113(9):1329-1338. doi:10.1038/s41395-018-0033-8 29523864

[48] Szabo SM, Bibby M, Yuan Y, . The epidemiologic burden of hepatitis C virus infection in Latin America. Ann Hepatol. 2012;11(5):623-635. doi:10.1016/S1665-2681(19)31435-8 22947522

[49] Wang T, Liu Y, Letran D, . Healthcare disparities identified between Hmong and other Asian origin groups living with chronic hepatitis B infection in Sacramento County 2014-2017. J Community Health. 2020;45(2):412-418. doi:10.1007/s10900-019-00763-1 31612369 PMC7489436

[50] Nguyen LH, Nguyen MH. Systematic review: Asian patients with chronic hepatitis C infection. Aliment Pharmacol Ther. 2013;37(10):921-936. doi:10.1111/apt.12300 23557103

[51] Younossi ZM, Golabi P, Paik JM, Henry A, Van Dongen C, Henry L. The global epidemiology of nonalcoholic fatty liver disease (NAFLD) and nonalcoholic steatohepatitis (NASH): a systematic review. Hepatology. 2023;77(4):1335-1347. doi:10.1097/HEP.0000000000000004 36626630 PMC10026948

[52] Centers for Disease Control and Prevention. Obesity, adult obesity facts. Accessed January 9, 2025. https://www.cdc.gov/obesity/adult-obesity-facts/index.html

[53] Sohn W, Lee HW, Lee S, . Obesity and the risk of primary liver cancer: a systematic review and meta-analysis. Clin Mol Hepatol. 2021;27(1):157-174. doi:10.3350/cmh.2020.0176 33238333 PMC7820201

[54] Shah NS, Luncheon C, Kandula NR, . Heterogeneity in obesity prevalence among Asian American adults. Ann Intern Med. 2022;175(11):1493-1500. doi:10.7326/M22-0609 36191316 PMC10323861

[55] Vicks WS, Lo JC, Guo L, . Prevalence of prediabetes and diabetes vary by ethnicity among U.S. Asian adults at healthy weight, overweight, and obesity ranges: an electronic health record study. BMC Public Health. 2022;22(1):1954. doi:10.1186/s12889-022-14362-8 36273116 PMC9587616

[56] DECODA Study Group. Prevalence of the metabolic syndrome in populations of Asian origin: comparison of the IDF definition with the NCEP definition. Diabetes Res Clin Pract. 2007;76(1):57-67. doi:10.1016/j.diabres.2006.07.020 17010470

[57] World Health Organization. Immunization data: hepatitis B vaccination coverage. Accessed August 26, 2024. https://immunizationdata.who.int/global/wiise-detail-page/hepatitis-b-vaccination-coverage

[58] Altekruse SF, Henley SJ, Cucinelli JE, McGlynn KA. Changing hepatocellular carcinoma incidence and liver cancer mortality rates in the United States. Am J Gastroenterol. 2014;109(4):542-553. doi:10.1038/ajg.2014.11 24513805 PMC4148914

[59] Lin SY, Chang ET, So SK. Why we should routinely screen Asian American adults for hepatitis B: a cross-sectional study of Asians in California. Hepatology. 2007;46(4):1034-1040. doi:10.1002/hep.21784 17654490

[60] LeFevre ML; U.S. Preventive Services Task Force. Screening for hepatitis B virus infection in nonpregnant adolescents and adults: U.S. Preventive Services Task Force recommendation statement. Ann Intern Med. 2014;161(1):58-66. doi:10.7326/M14-1018 24863637

[61] Jensen TS, Chin J, Ashby L, Paserchia L, Issa M. Centers for Medicare & Medicaid Services. Screening for hepatitis B virus (HBV) infection (CAG-00447N). Accessed August 26, 2024. https://www.cms.gov/medicare-coverage-database/view/ncacal-decision-memo.aspx?proposed=N&NCAId=283

[62] Chao DT, Abe K, Nguyen MH. Systematic review: epidemiology of hepatitis C genotype 6 and its management. Aliment Pharmacol Ther. 2011;34(3):286-296. doi:10.1111/j.1365-2036.2011.04714.x 21623850

[63] Zhang Y, Gao Z, Wang S, . Hepatitis C virus genotype/subtype distribution and evolution among Chinese blood donors: revealing recent viral expansion. PLoS One. 2020;15(7):e0235612. doi:10.1371/journal.pone.0235612 32649673 PMC7351211

