# Supplementary material for: Liver Cancer Etiology in Asian Subgroups and American Indian, Black, Latino, and White Populations
Source: JAMA Netw Open. 2025 Mar 27;8(3):e252208. doi: 10.1001/jamanetworkopen.2025.2208 (PMC11950898; doi:10.1001/jamanetworkopen.2025.2208)
Supplement: Supplement 1. — eFigure. Age-Specific Incidence Rate for HCV-HCC With Birth Cohort Information eTable 1. Diagnostic Code Definitions for HCC California Data, 2010-2018 eTable 2. Patient Region of Residence Defined by County of Residence at the Time of HCC Diagnosis eTable 3. Stage at Diagnosis by Detailed Race/Ethnicity of HCC Cases eTable 4. Insurance Type by Detailed Race/Ethnicity of HCC Cases eTable 5. Age-Adjusted Incidence Rates per 100,000 and Temporal Trends Annual Percent Change for HCC by Detailed Race/Ethnicity and Sex eTable 6. Temporal Trends Annual Percent Change for HCC by Age Group and Etiology [file jamanetwopen-e252208-s001.pdf]

Supplemental Online Content

Pinheiro PS, Zhang J, Setiawan VW, Cranford HM, Wong RJ, Liu L. Liver cancer etiology in Asian subgroups and American Indian, Black, Latino, and White populations. *JAMA Netw Open*. 2025;8(3):e252208. doi:10.1001/jamanetworkopen.2025.2208

eFigure. Age-Specific Incidence Rate for HCV-HCC With Birth Cohort Information

eTable 1. Diagnostic Code Definitions for HCC California Data, 2010-2018

eTable 2. Patient Region of Residence Defined by County of Residence at the Time of HCC Diagnosis

eTable 3. Stage at Diagnosis by Detailed Race/Ethnicity of HCC Cases

eTable 4. Insurance Type by Detailed Race/Ethnicity of HCC Cases

eTable 5. Age-Adjusted Incidence Rates per 100,000 and Temporal Trends Annual Percent Change for HCC by Detailed Race/Ethnicity and Sex

eTable 6. Temporal Trends Annual Percent Change for HCC by Age Group and Etiology

This supplemental material has been provided by the authors to give readers additional information about their work.

© 2025 Pinheiro PS et al. *JAMA Network Open*.

Supplementary Figure 1. Age-specific incidence rate (per 100,000) for HCV-HCC with birth cohort information. California, 2008-2018. A. Males by race/ethnicity; B. Females by race/ethnicity. *Abbreviations: AI, American Indian; API, Asian/Pacific Islander; HCC, hepatocellular carcinoma; NL, non-Latino; US, United States.*

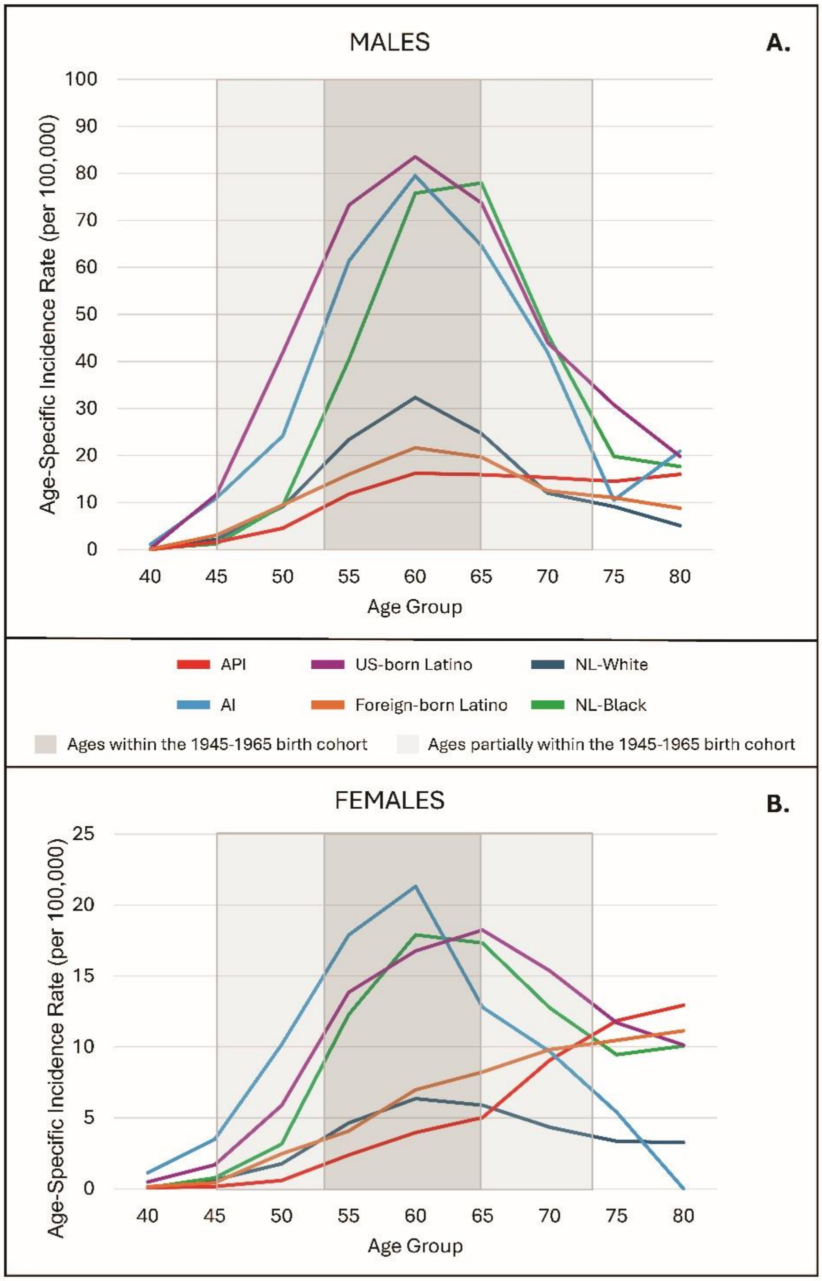

© 2025 Pinheiro PS et al. *JAMA Network Open*.

SUPPLEMENTARY TABLES:

Supplementary Table 1. Diagnostic code definitions for HCC California data, 2010–2018.		
**Category**	**ICD-9-CM Codes**	**ICD-10-CM Codes**
**HCC Etiology**		
**Chronic Hepatitis C Virus Infection**	070.41, 070.44, 070.51, 070.54, 070.7, V02.62, 070.70, 070.71	B17.10, B17.11, B18.2, B19.20, B19.21
**Chronic Hepatitis B Virus Infection**	070.20, 070.22, 070.23, 070.30, 070.31, 070.32, 070.33, 070.42, 070.52, V02.61, 070.21	B16.X, B17.0, B18.0, B18.1, B19.1X
**Alcohol-Related Liver Conditions**	571.0, 571.1, 571.2, 571.3, 291, 291.0-291.5, 291.8, 291.81, 291.82, 291.89, 291.9, 303, 303.0, 303.00-303.03, 303.9, 303.90-303.93, 305.0, 305.00-305.03, 357.5, 425.5, 535.3, 535.30, 535.31, 790.3, 980, 980.0, 980.8, 980.9, V11.3, V79.1	F10.X, F10.XX, F10.XXX, G62.1, I42.6, K29.2X, K70.X, K70.XX, R78.0, T51.0X1, T51.0X2, T51.0X3, T51.0X4, T51.8X1, T51.8X2, T51.8X3, T51.8X4, T51.91X, T51.92X, T51.93X, T51.94X
**Metabolic/MASLD-Related Conditions**	250.00, 250.02, 250.10, 250.12, 250.20, 250.22, 250.30, 250.32, 250.40, 250.42, 250.50, 250.52, 250.60, 250.62, 250.70, 250.72, 250.80, 250.82, 250.90, 250.92, 272.0, 272.1, 272.2, 272.4, 272.5, 272.7, 272.9, 277.7, 278, 278.0, 278.00-278.03, 278.1, 278.8, 401-404, 571.8, 783.1, 790.2, 790.21, 790.22, 790.29, V45.86, V77.8, V85.4, V85.30-V85.45, 401.X - 404X , 401.XX - 404.XX	E11.X, E11.XX, E11.XXX, E13.X, E13.XX, E13.XXX, E66.X, E66.XX, E67.0, E67.8, E68.0, E75.21, E75.22, E75.249, E77.0, E77.1, E78.X, E78.XX, E88.81X, I10.0, I11.X, I12.X, I13.X, I13.XX, I15.X, I16.X, K75.81, K76.0, K76.89, R63.5, R73.X, R73.XX, Z68.3X, Z68.4X, Z98.84, I10
**Autoimmune Conditions**	242.40, 242.41, 242.80, 242.81, 242.90, 242.91, 250.01, 250.03, 250.11, 250.13, 250.21, 250.23, 250.31, 250.33, 250.41, 250.43, 250.51, 250.53, 250.61, 250.63, 250.71, 250.73, 250.81, 250.83, 250.91, 250.93, 274.00-274.03, 274.10, 274.19, 274.81, 274.82, 274.89, 274.90, 555.0, 555.1, 555.2, 555.9, 556.0-556.9, 571.42, 577.1, 579.0, 710.0, 711.10-711.19, 714.0, 714.2, 714.30-714.33	E05.3X, E05.4X, E05.8X, E05.9X, E10.1X, E10.21, E10.29, E10.31X, E10.36, E10.37, E10.39, E10.40, E10.51, E10.618, E10.62X, E10.63X, E10.64X, E10.65, E10.69, E10.8, E10.9, K50.00, K50.10, K50.80, K50.90, K51.00, K51.20, K51.30, K51.40, K51.50, K51.80, K51.90, K75.2- K75.4, K86.1, K90.0, M02.3X, M02.3XX, M05.3X, M05.3XX, M05.6X, M05.6XX, M06.X, M06.XX, M06.XXX, M08.X, M08.XX, M08.XXX, M10.00, M10.30, M10.40, M10.9, M1A.00X1, M1A.20X1, M1A.30X1, M1A.40X1, M1A.9XX0, M1A.9XX1, M32.10, N20.0
**Genetic Conditions**	270.2, 271.0, 273.4, 275.0, 275.01, 275.1, 277.1	E70.21, E70.29, E70.30, E70.5, E70.8, E74.00, E74.01, E74.04, E74.09, E74.4, E80.0, E80.20, E80.29, E83.0X, E83.11X, E88.01
**Cryptogenic**	571.42, 576.1, 275.01	K75.4, K83.0, K83.01, E83110
**Cirrhosis**		
**Cirrhosis**	275.01 (hereditary hemochromatosis), 456, 456.0-456.2, 456.21(esophageal varices), 571.2 (Alcoholic cirrhosis of liver), 571.5 (Cirrhosis of liver without mention of alcohol), 571.6 (Biliary cirrhosis), 572.3 (Portal hypertension), 572.4 (Hepatorenal syndrome), 777.8 (Other specified perinatal disorders of digestive system), 789.5 (Ascites), 789.51 (Malignant ascites), 789.59 (Other ascites)	E83.110 (Hereditary hemochromatosis), I85.X, I85.XX (Esophageal varices), K70.3X (Alcoholic cirrhosis of liver), K71.7 (Toxic liver disease with fibrosis and cirrhosis of liver), K74.3-K74.5 (Biliary cirrhosis), K74.6X (Unspecified cirrhosis of liver), K76.6 (Portal hypertension), K76.7 (Hepatorenal syndrome), K76.82 (Hepatic encephalopathy), K70.11 (Alcoholic hepatitis with ascites), K70.31 (Alcoholic cirrhosis of liver with ascites), K71.51 (Toxic liver disease with chronic active hepatitis with ascites), R18.0 (Ascites), R18.8 (Other ascites)
Abbreviations: HCC, hepatocellular carcinoma; ICD-9-CM, International Classification of Diseases, Ninth Revision, Clinical Modification; ICD-10-CM, International Classification of Diseases, Tenth Revision, Clinical Modification; MASLD, metabolic dysfunction-associated steatotic liver disease.		

© 2025 Pinheiro PS et al. *JAMA Network Open*.

Supplementary Table 2. Patient region of residence defined by county of residence at the time of HCC diagnosis.

Category	Counties
**Northern**	Monterey, San Benito, Santa Clara, Santa Cruz, Alpine, Amador, Calaveras, El Dorado, Nevada, Placer, Sacramento, San Joaquin, Sierra, Solano, Sutter, Yolo, Yuba, Butte, Colusa, Del Norte, Glenn, Humboldt, Lake, Lassen, Mendocino, Modoc, Napa, Plumas, Shasta, Siskiyou, Sonoma, Tehama, Trinity, Alameda, Contra Costa, Marin, San Francisco, San Mateo.
**Southern**	San Luis Obispo, Santa Barbara, Ventura, San Diego, Los Angeles, Orange.
**Inland**	Fresno, Kern, Kings, Madera, Mariposa, Merced, Stanislaus, Tulare, Tuolumne, Inyo, Mono, Riverside, San Bernardino, Imperial.

Abbreviations: HCC, hepatocellular carcinoma.

© 2025 Pinheiro PS et al. *JAMA Network Open*.

Supplementary Table 3. Stage at diagnosis by detailed race/ethnicity[a] of HCC cases. California, 2010–2018.								
**Level I Race/ethnicity**	**Level II Race/ethnicity**		**Total N**	**Localized n (%[b])**	**Regional n (%[b])**	**Remote n (%[b])**	**Unknown n (%[b])**
**American Indian**			413	197 (47.7)	93 (22.5)	60 (14.5)	63 (15.3)
**Asian/Pacific Islander[c]**			6,561	3,424 (52.2)	1,501 (22.9)	812 (12.4)	824 (12.6)
	Chinese		1,713	933 (54.5)	331 (19.3)	190 (11.1)	259 (15.1)
	Filipino		1,167	562 (48.2)	298 (25.5)	163 (14.0)	144 (12.3)
	Japanese		305	163 (53.4)	74 (24.3)	35 (11.5)	33 (10.8)
	Korean		628	308 (49.0)	152 (24.2)	78 (12.4)	90 (14.3)
	Pacific Islander		201	111 (55.2)	51 (25.4)	24 (11.9)	15 (7.5)
	South Asian[d]		257	134 (52.1)	64 (24.9)	41 (16.0)	18 (7.0)
	Vietnamese		1,634	892 (54.6)	376 (23.0)	184 (11.3)	182 (11.1)
	Other Southeastern Asian[e]		455	202 (44.4)	111 (24.4)	77 (16.9)	65 (14.3)
		Cambodian	193	87 (45.1)	51 (26.4)	29 (15.0)	26 (13.5)
		Hmong	47	22 (46.8)	x	x	x
		Laotian	128	52 (40.6)	32 (25.0)	26 (20.3)	18 (14.1)
		Thai	87	41 (47.1)	18 (20.7)	14 (16.1)	14 (16.1)
**Latino[c]**			9,992	4,951 (49.5)	2,310 (23.1)	1,286 (12.9)	1,445 (14.5)
	US-born		5,097	2,516 (49.4)	1,184 (23.2)	655 (12.9)	741 (14.5)
	Foreign-born		4,895	2,435 (49.7)	1,126 (23.0)	631 (12.9)	704 (14.4)
**Non-Latino Black**			2,419	1,103 (45.6)	590 (24.4)	346 (14.3)	380 (15.7)
**Non-Latino White**			12,190	5,860 (48.1)	2,896 (23.8)	1,609 (13.2)	1,825 (15.0)

a. Small number of observations not shown for confidentiality protection and indicated with x; b. Percentage calculated based on row total; c. All race-ethnicities combined only includes those listed here (excludes multiracial, other, and unknown race); d. Includes all cases of races related to Bangladesh, India, Nepal, Pakistan, and Sri Lanka; e. Includes all cases of races related to Cambodia, Laos, and Thailand, plus Hmong people.

Abbreviations: HCC, hepatocellular carcinoma; N, number; US, United States.

© 2025 Pinheiro PS et al. *JAMA Network Open*.

Supplementary Table 4. Insurance type by detailed race/ethnicity[a] of HCC cases. California, 2010–2018.									
**Level I Race/ethnicity**	**Level II Race/ethnicity**		**Total N**	**Medicaid n (%[b])**	**Medicare n (%[b])**	**Private n (%[b])**	**Uninsured n (%[b])**	**Unknown n (%[b])**
**American Indian**			413	85 (20.6)	158 (38.3)	121 (29.3)	x	x
**Asian/Pacific Islander[c]**			6,561	1,151 (17.5)	2,624 (40.0)	2,237 (34.1)	127 (1.9)	422 (6.4)
	Chinese		1,713	271 (15.8)	640 (37.4)	663 (38.7)	19 (1.1)	120 (7.0)
	Filipino		1,167	166 (14.2)	466 (39.9)	445 (38.1)	21 (1.8)	69 (5.9)
	Japanese		305	15 (4.9)	152 (49.8)	110 (36.1)	x	x
	Korean		628	95 (15.1)	282 (44.9)	166 (26.4)	20 (3.2)	65 (10.4)
	Pacific Islander		201	38 (18.9)	76 (37.8)	70 (34.8)	x	x
	South Asian[d]		257	63 (24.5)	99 (38.5)	78 (30.4)	x	x
	Vietnamese		1,634	331 (20.3)	697 (42.7)	494 (30.2)	22 (1.3)	90 (5.5)
	Other Southeastern Asian[e]		455	123 (27.0)	150 (33.0)	133 (29.2)	12 (2.6)	37 (8.1)
		Cambodian	193	60 (31.1)	66 (34.2)	49 (25.4)	x	x
		Hmong	47	15 (31.9)	16 (34.0)	11 (23.4)	x	x
		Laotian	128	24 (18.8)	47 (36.7)	37 (28.9)	x	x
		Thai	87	24 (27.6)	21 (24.1)	36 (41.4)	x	x
**Latino[c]**			9,992	2,250 (22.5)	3,704 (37.1)	3,040 (30.4)	226 (2.3)	772 (7.7)
	US-born		5,097	1,051 (20.6)	1,880 (36.9)	1,672 (32.8)	85 (1.7)	408 (8.0)
	Foreign-born		4,895	1,199 (24.5)	1,824 (37.3)	1,368 (28.0)	141 (2.9)	364 (7.4)
**Non-Latino Black**			2,419	476 (19.7)	979 (40.5)	716 (29.6)	45 (1.9)	203 (8.4)
**Non-Latino White**			12,190	1,547 (12.7)	5,354 (43.9)	4,030 (33.1)	189 (1.6)	1,070 (8.8)

a. Small number of observations not shown for confidentiality protection and indicated with x; b. Percentage calculated based on row total; c. All race-ethnicities combined only includes those listed here (excludes multiracial, other, and unknown race); d. Includes all cases of races related to Bangladesh, India, Nepal, Pakistan, and Sri Lanka; e. Includes all cases of races related to Cambodia, Laos, and Thailand, plus Hmong people.

Abbreviations: HCC, hepatocellular carcinoma; N, number; US, United States.

© 2025 Pinheiro PS et al. *JAMA Network Open*.

Supplementary Table 5. Age-adjusted[a] incidence rates (AAIR) per 100,000 and temporal trends annual percent change (APC) for HCC by detailed race/ethnicity and sex. California, 2010–2018.					
**Level I Race/ethnicity**	**Level II Race/ethnicity**	**AAIR Trends**		**All HCC Cases**	
		**2010–2011**	**2017–2018**	**Trend Period**	**APC (95%CI)**
**MALES**					
**Asian/Pacific Islander[b]**		20.2	14.6	2010–2018	-4.1 (-5.7 to -2.5)
	Chinese	19.9	14.3	2010–2018	-4.6 (-6.0 to -3.2)
	Filipino	16.1	11.2	2010–2018	-5.2 (-9.6 to -0.5)
	Japanese	10.6	7.0	2010–2018	-4.1 (-11.3 to + 3.8)
	Korean	26.7	14.6	2010–2018	-7.9 (-13.7 to -2.4)
	Pacific Islander	17.6	22.5	2010–2018	+3.6 (-3.1 to +10.8)
	South Asian[c]	8.3	8.2	2010–2018	+0.5 (-4.8 to +7.0)
	Vietnamese	47.8	34.8	2010–2018	-4.1 (-7.1 to -1.0)
	Other Southeastern Asian[d]	51.7	32.1	2010–2018	-6.2 (-12.4 to +0.7)
**Latino[b]**		18.9	17.7	2010–2018	-0.9 (-1.6 to -0.1)
	US-born	29.3	24.1	2010–2018	-2.7 (-4.6 to -0.8)
	Foreign-born	12.7	13.6	2010–2018	+1.3 (-0.1 to +2.7)
**FEMALES**					
**Asian/Pacific Islander[b]**		6.7	4.4	2010–2018	-5.5 (-7.7 to -3.3)
	Chinese	6.3	3.9	2010–2018	-6.4 (-9.6 to -3.2)
	Filipino	5.4	4.4	2010–2018	-3.8 (-12.6 to +6.2)
	Japanese	4.8	3.1	2010–2018	-6.5 (-12.9 to -0.1)
	Korean	7.9	5.4	2010–2018	-6.5 (-11.4 to -1.5)
	Pacific Islander	8.6	8.9	2010–2018	+0.7 (-13.6 to +18.0)
	South Asian[c]	3.3	1.5	2010–2018	-5.3 (-16.5 to +7.6)
	Vietnamese	14.9	8.0	2010–2018	-8.1 (-12.8 to -3.1)
	Other Southeastern Asian[d]	14.9	8.0	2010–2018	-6.6 (-17.2 to +5.1)
**Latino[b]**		6.1	6.8	2010–2018	+1.1 (-1.5 to +4.0)
	US-born	6.7	7.4	2010–2018	+1.4 (-1.2 to +4.1)
	Foreign-born	5.6	6.2	2010–2018	+0.4 (-2.6 to +3.8)

a. Age-adjusted to the 2000 US standard population; b. Includes all cases of this race and/or ethnicity, not just listed groups; c. Includes all cases of races related to Bangladesh, India, Nepal, Pakistan, and Sri Lanka; d. Includes all cases of races related to Cambodia, Laos, and Thailand, plus Hmong people. *Abbreviations: AAIR, age-adjusted incidence rate; APC, annual percent change; CI, confidence interval; HCC, hepatocellular carcinoma; US, United States.*

© 2025 Pinheiro PS et al. *JAMA Network Open*.

Supplementary Table 6. Temporal trends annual percent change (APC) for HCC by age group and etiology. California, 2010–2018.

Age Group	Trend Period	Total[a]	HCV	HBV	MASLD	ALD
		APC (95%CI)				
<49	2010–2018	-6.0 (-9.6 to -2.5)	-12.4 (-18.6 to -7.0)	-4.8 (-10.8 to +1.1)	+2.1 (-5.0 to +9.4)	+2.5 (-6.7 to +12.4)
50-69	2010–2014	+2.3 (+0.6 to +4.6)	+2.6 (+1.2 to +4.2)	-2.7 (-4.7 to -0.6)	+3.3 (+2.0 to +4.6)	+2.6 (+0.7 to +4.7)
	2014–2018	-4.6 (-6.8 to -3.2)	-7.5 (-8.9 to -6.1)			
70+	2010–2018	+0.5 (-1.1 to +2.1)	-1.0 (-3.3 to +1.5)	-2.8 (-5.8 to +0.4)	+2.2 (+1.2 to +3.4)	+1.7 (0.0 to +3.6)

a. Includes all listed as well as Cryptogenic and Others (e.g. genetic, auto-immune). *Abbreviations: ALD, alcoholic liver disease; APC, annual percent change; CI, confidence interval; HCC, hepatocellular carcinoma; HCV, hepatitis C virus; HBV, hepatitis B virus; MASLD, metabolic dysfunction-associated steatotic liver disease;.*

© 2025 Pinheiro PS et al. *JAMA Network Open*.
