# Supplementary material for: Liver Cancer Etiology in Asian Subgroups and American Indian, Black, Latino, and White Populations
Source: JAMA Netw Open. 2025 Mar 27;8(3):e252208. doi: 10.1001/jamanetworkopen.2025.2208 (PMC11950898; doi:10.1001/jamanetworkopen.2025.2208)
Supplement: Supplement 2. — Data Sharing Statement [file jamanetwopen-e252208-s002.pdf]

Data Sharing Statement

Pinheiro. Liver Cancer Etiology in Asian Subgroups and American Indian, Black, Latino, and White Populations. *JAMA Netw Open*. Published March 27, 2025.
doi:10.1001/jamanetworkopen.2025.2208

Data
Data available: No

Additional Information
Explanation for why data not available: The data underlying this article are available for specific use upon approvals from the California and Los Angeles County Cancer Surveillance Programs after required authorization and appropriate Institutional Review Board approval.
